# Exposing Pathological Sensory Predictions in Tinnitus Using Auditory Intensity Deviant Evoked Responses

**DOI:** 10.1523/JNEUROSCI.1308-19.2019

**Published:** 2019-12-11

**Authors:** William Sedley, Kai Alter, Phillip E. Gander, Joel Berger, Timothy D. Griffiths

**Affiliations:** ^1^Newcastle University Medical School, Newcastle upon Tyne NE2 4HH, United Kingdom, and; ^2^Human Brain Research Laboratory, University of Iowa, Hospitals and Clinics, Iowa City, Iowa 52242

**Keywords:** biomarker, electroencephalography, mismatch negativity, phantom perception, predictive coding, tinnitus

## Abstract

We tested the popular, unproven theory that tinnitus is caused by resetting of auditory predictions toward a persistent low-intensity sound. Electroencephalographic mismatch negativity responses, which quantify the violation of sensory predictions, to unattended tinnitus-like sounds were greater in response to upward than downward intensity deviants in 26 unselected chronic tinnitus subjects with normal to severely impaired hearing, and in 15 acute tinnitus subjects, but not in 26 hearing and age-matched controls (*p* < 0.001, receiver operator characteristic, area under the curve, 0.77), or in 20 healthy and hearing-impaired controls presented with simulated tinnitus. The findings support a prediction resetting model of tinnitus generation, and may form the basis of a convenient tinnitus biomarker, which we name Intensity Mismatch Asymmetry, which is usable across species, is quick and tolerable, and requires no training.

**SIGNIFICANCE STATEMENT** In current models, perception is based around the generation of internal predictions of the environment, which are tested and updated using evidence from the senses. Here, we test the theory that auditory phantom perception (tinnitus) occurs when a default auditory prediction is formed to explain spontaneous activity in the subcortical pathway, rather than ignoring it as noise. We find that chronic tinnitus patients show an abnormal pattern of evoked responses to unexpectedly loud and quiet sounds that both supports this hypothesis and provides fairly accurate classification of tinnitus status at the individual subject level. This approach to objectively demonstrating the predictions underlying pathological perceptual states may also have a much wider utility, for instance, in chronic pain.

## Introduction

**Figure 1.**
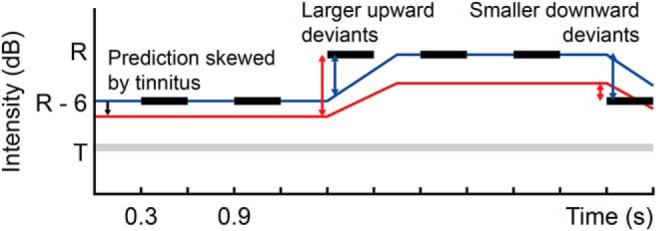
Experimental hypothesis and paradigm. The paradigm is a roving MMN paradigm, with 300 ms pure tones (black bars) interspersed with 300 ms intervals. Stimulus frequency is matched to within or adjacent to the individual tinnitus frequency band. Intensity is the roved parameter, between 0 and −6 dB relative to an individualized reference intensity (R). We hypothesized that control subjects would make optimal predictions of upcoming stimuli (blue line) based on recent stimulus history, the presence of a tinnitus prediction (T and gray line) would result in tinnitus subjects forming an intermediate prediction (red line) between the optimal stimulus-based prediction and the tinnitus prediction. This would result in an asymmetry between MMN responses to upward and downward intensity deviants in tinnitus subjects (red arrows) compared with controls (blue arrows). Many aspects of stimulus sequences are predicted, but illustrated predictions here refer only to the intensity of the next upcoming stimulus. Because control subjects' default prediction is of no sound at all, rather than an auditory percept (e.g., tinnitus) but with zero intensity, this does not impact the predicted intensity of upcoming stimuli.

Tinnitus, the persistent perception of an illusory sound, affects 13%, and significantly impairs the quality of life of 2%, of the population (Shargorodsky et al., 2010). The search for effective treatments is greatly hampered by limited understanding of its mechanisms. Its major risk factor is hearing loss, which leads to increased central gain (i.e., increased firing rate and/or synchrony in for a given input) (Gold and Bajo, 2014), although a review of current evidence suggests that these changes may be contributory to tinnitus, but not sufficient to cause it (Sedley, 2019), while other evidence suggests that gain increases may be irrelevant, or even protective, with respect to tinnitus (Rüttiger et al., 2013; Singer et al., 2013; Hofmeier et al., 2018), and the presence or absence of hyperacusis can confound results (Gu et al., 2010; Möhrle et al., 2019). We have recently proposed a theory of tinnitus causation, which shares some features with an earlier theory (De Ridder et al., 2014a), in which a crucial process is the learning of a default “tinnitus prediction” by higher perceptual centers (Sedley et al., 2016). Specifically, we suggested that the origin of the tinnitus signal is spontaneous firing in the ascending auditory pathway, but that this is usually successfully ignored as irrelevant noise. Furthermore, we proposed that, once the brain has recognized the tinnitus signal as a sound source, it forms a default prediction of that sound continuing, which prevents the spontaneous activity being ignored as noise, and that prediction ensures the persistence of tinnitus once present for a sufficient length of time. Similar predictive coding (Rao and Ballard, 1999; Friston and Kiebel, 2009) models have been proposed for many perceptual disorders (Edwards et al., 2012; Adams et al., 2013; Kumar et al., 2014), but actually demonstrating the aberrant predictions themselves is a much greater challenge; hence, these models rely on circumstantial evidence. The present work aimed to search for evidence of the existence of a default auditory prediction that might underpin chronic tinnitus. We focused on the mismatch negativity (MMN) (Näätänen and Alho, 1995; Näätänen et al., 2007) evoked response, which occurs across many sensory modalities in response to stimuli that differ (e.g., in frequency or intensity) from a series of preceding stimuli. Moreover, MMN magnitude quantitatively indicates the extent to which a particular stimulus violates a prior prediction of what that stimulus will be (Garrido et al., 2013), making it a useful tool for inferring the content of sensory predictions. In this study, we compared MMN responses, obtained from a roving oddball paradigm (Garrido et al., 2008) featuring pure tones close to the tinnitus frequency, to upward and downward intensity deviants to expose alterations of auditory predictions that might be associated with tinnitus (Fig. 1). Specifically, because tinnitus is a quieter sound than those used in the experiment, we hypothesized that the tinnitus prediction should skew predictions of intensity downward, meaning that downward intensity deviants should produce smaller MMN responses, and upward intensity deviants produce larger responses, compared with matched controls. Although there have been numerous MMN studies in tinnitus (Weisz et al., 2004; Holdefer et al., 2013; Mahmoudian et al., 2013; El-Minawi et al., 2018; Mohebbi et al., 2019a), the present study differs importantly in that it both targets the tinnitus frequency and features deviants in intensity, and thus is uniquely able to address this hypothesis. Our results showed a striking asymmetry of MMN responses of exactly the type predicted, compared with age- and hearing-matched controls. This supports the prediction hypothesis of tinnitus (though other interpretations are possible), and can classify individual subjects' tinnitus status with a fair degree of accuracy. The work thus introduces a new potential tinnitus biomarker for further human and animal work.

## Materials and Methods

### 

#### 

##### Subjects.

**Figure 2.**
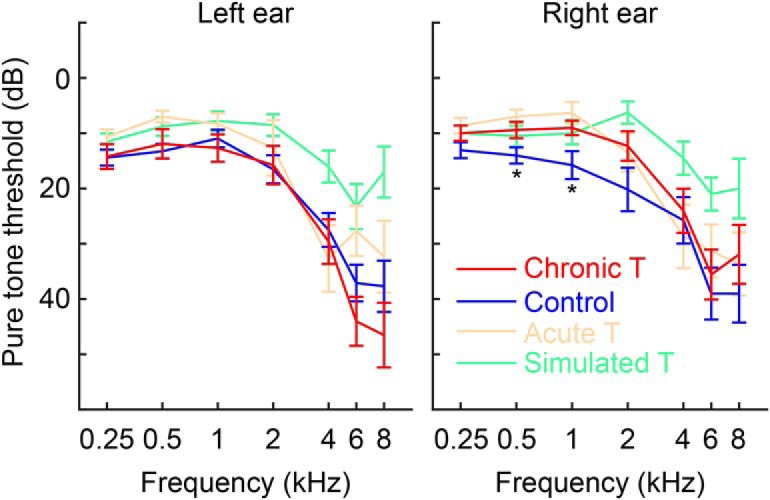
Pure tone audiometry of subject groups. Plots indicate group mean and SE at each frequency/ear. *p < 0.05, differences between chronic tinnitus (T) subjects and matched controls significant at uncorrected, which were only present at frequencies remote from any experimental stimuli used.

Unselected chronic tinnitus subjects (*n* = 26) were recruited from local research volunteer mailing lists, with the only inclusion criteria being age 18 or over, persistent tinnitus for longer than 6 months, ability to perform experiments, and absence of structural brain pathology or profound hearing loss in the tinnitus ear(s). Nontinnitus controls (matched *n* = 26, and simulated tinnitus *n* = 20) were recruited from the same lists and subjected to pure-tone audiometry, with the best matches being invited to take part in the full study on a separate occasion. Acute tinnitus subjects (*n* = 15) were recruited via paid advertising on an Internet search engine, with the same inclusion criteria as for chronic subjects, but with tinnitus duration of <6 weeks. Group sizes were chosen as the minimum necessary to give a high chance of demonstrating the predicted effects. Subject characteristics can be found in Table 1 of the main text. No significant differences in hearing thresholds (Fig. 2) at any frequency were present between chronic tinnitus subjects and matched controls, except at 0.5 and 1 kHz in the right ear, which were remote from the stimulus and tinnitus frequencies (*p* < 0.05).

**Table 1.** Subject, tinnitus, and stimulus characteristics*^a^*

	Chronic T	Control	*p* (T vs C)	Acute T	Simulated T
Demographics					
Age	55.4 (13.6)	59.7 (15.3)	0.31	53.8 (12.5)	45.0 (19.1)
Sex	13 F/13 M	19 F/7 M	0.014*	6 F/9 M	10 F/10 M
Tinnitus characteristics					
Duration	15.5 (16.7) years	—	—	4.2 (1.7) weeks	—
THI	31 (28.1)	—	—	27 (23.2)	—
T ear	11/15/0 L/C/R	—	—	4/8/3 L/C/R	—
T character	13/13 T/N	—	—	9/6 T/N	—
VAS loudness	5.0 (2.1)	—	—	4.8 (1.9)	—
VAS distress	4.8 (2.9)	—	—	4.8 (2.8)	—
% awareness	55.2 (34.3)	—	—	44.6 (23.7)	—
T match CF (Hz)	6777 (2009)	—	—	7047 (2536)	—
T match BW (oct)	0.25 (0.25)	—	—	0.17 (0.20)	—
Stimulus and hearing characteristics					
Center F (Hz)	7709 (2706)		—	7582 (2517)	7164 (2861)
Edge F (Hz)	5901 (1948)		—	6028 (2418)	—
Edge F to center F (oct)	0.37 (0.27)		—	0.39 (0.27)	—
Edge F to match lower bound (oct)	0.078 (0.27)	—	—	0.18 (0.18)	—
Thresh center (dB)	44.8 (26.1)	37.9 (21.1)	0.19	34.3 (20.6)	20.3 (19.6)
Thresh edge (dB)	40.3 (21.1)	35.4 (17.0)	0.25	31.6 (22.7)	—
SPL center (dB)	78.1 (18.4)	78.2 (11.2)	0.99	79.5 (13.0)	79.0 (14.1)
SPL edge (dB)	72.8 (16.4)	78.9 (9.68)	0.043*	76.3 (12.6)	—
SL center (dB)	33.3 (23.3)	40.2 (20.6)	0.15	45.2 (23.6)	58.7 (20.6)
SL edge (dB)	32.5 (20.6)	43.4 (18.3)	0.012*	44.7 (26.8)	—

*^a^*Values inside and outside parentheses indicate mean and SD, respectively, unless otherwise indicated. T, Tinnitus; C, control; VAS, visual analog scale; CF, center frequency; BW, bandwidth; Oct, octaves; Thresh, hearing threshold (at specified frequency); SPL, sound pressure level (of stimulus); SL, sensation level (of stimulus); L/C/R, left (predominant)/center/right (predominant); T/N, tonal/narrowband noise; —, not applicable to particular group.

*p < 0.05.

Recruitment and data collection occurred between November 2017 and October 2018, with additional simulated tinnitus data being collected in August 2019. The study was given a favorable opinion by the Newcastle University Research Ethics Committee, and all participants provided written informed consent according to the Declaration of Helsinki.

##### Clinical and psychophysical assessment.

All research activity occurred within the Institute of Neuroscience, Newcastle University. Subjects completed a short questionnaire covering demographic details, health conditions, and medications. Tinnitus subjects also indicated the duration, character, and laterality of their tinnitus, along with visual analog scale ratings of their average and current tinnitus loudness, average annoyance, and completed the Tinnitus Handicap Inventory (THI) (Newman et al., 1996). All subjects underwent pure tone audiometry at octave intervals from 0.25 to 8 kHz, with the addition of 6 kHz.

Tinnitus subjects performed five rounds of tinnitus matching, using custom-made tools in MATLAB (The MathWorks), based on interactively tuning narrowband noise with a Hanning-shaped spectrum in terms of its center frequency, bandwidth, intensity, and laterality balance. Each round used random starting parameters for visual analog scale and bandwidth. Subjects were allowed to discard matches they regarded as suboptimal, and the mean visual analog scale and bandwidth across remaining matches were used as a starting point for stimulus generation. Control subjects used their matched tinnitus-subject's data for stimulus generation.

For each subject, two pure tone stimuli of different frequencies were created: one at the tinnitus match center and one at the lower edge of the Hanning passband. Tinnitus subjects had one opportunity to tune the frequency of these until perceived as in the center of the tinnitus frequency band (“center”), and the other as close as possible to the tinnitus frequency while being distinctly lower in frequency (“edge”). The rationale for using these frequencies was to test whether any tinnitus-related effects were tightly locked to the tinnitus frequency, and whether precise tinnitus frequency matching would be required to observe these effects. All subjects then iteratively tuned the following parameters in sequence, until a full round passed with no further changes: laterality balance (center then edge), balancing the subjective loudness of both frequencies, and tuning overall intensity of both frequencies. Control subjects were allocated the stimulus frequencies of the tinnitus subject with the closest audiometric thresholds in stimulus ear/frequency, and were asked to make stimuli as loud as possible without resulting in even minor discomfort, or producing distortions from the headphones. The additional constraint for tinnitus subjects was that tinnitus must remain audible in the gaps between stimuli (i.e., tinnitus not be totally attenuated by residual inhibition).

##### Experimental design.

In a soundproof room, 64 channel EEG was recorded from participants, using an Activetwo system (Biosemi), while they were passively presented with the experimental stimuli through Sennheiser HD 380 pro headphones, and watched a silent subtitled movie. Electrode offset (equivalent to impedance) was kept within manufacturer-recommended limits of ±40 mV.

The paradigm was a roving MMN paradigm (Garrido et al., 2009), in which 300 ms pure tones (with 10 ms onset/offset ramps) were presented isochronously with a stimulus onset asynchrony of 600 ms. Stimuli were presented to the tinnitus ear if entirely or mainly unilateral (including to the matched control), and bilaterally in other cases. The roved parameter was stimulus intensity, which randomly alternated between 0 and −6 dB (relative to the subject-calibrated intensity) every four to eight stimuli. One block of the experiment comprised 21 such intensity changes, and a total of 50 blocks were presented, alternating between the center and edge frequency. A control deviant condition was superimposed on these sequences, whereby 1 in 10 stimuli (1/6 probability, after minimum separation of four stimuli) were duration deviants of 150 ms.

Simulated tinnitus subjects were simultaneously presented with ongoing narrowband noise on alternate blocks (“tinnitus on” condition). This noise had the spectrum of the tinnitus match of the acute or chronic tinnitus subject with the closest hearing thresholds at frequencies adjacent to their tinnitus frequency. To prevent the nonrepresentative scenario of the stimuli and “tinnitus” being perceptually identical, the bandwidth for simulated pure tone tinnitus was set to 1/40 octave. Intensity of the noise stimulus was set at the tinnitus match intensity initially, and the subject was asked to adjust the intensity, if necessary, to ensure it was loud enough to be audible over quiet speech, and quiet enough to not prevent a normal volume conversation. Such adjustments were not required in most cases.

##### EEG data processing.

Data analysis was performed in MATLAB, using the FieldTrip toolbox (Oostenveld et al., 2011). EEG data were recorded at 1024 Hz, downsampled to 256 Hz, and high-pass filtered from 0.3 Hz. Data were rereferenced to combined P9/P10, roughly corresponding to M1 and M2 locations. Bad channels were identified visually and reconstructed by interpolation. Data were epoched between −0.5 and 1 s peristimulus time, with demeaning and detrending. Epochs with grossly outlying maximum amplitudes, based on visual inspection, were excluded, followed by removal of ocular and muscle artifacts using independent component analysis. A mean of 23 components was removed per subject, with no significant difference in the number of components removed between tinnitus and control groups. Epochs were baseline corrected to −100–0 ms peristimulus time, and each epoch was summarized by four values derived from its (normalized within channel) time series: largest absolute amplitude in any channel at any time point; largest mean absolute deviation across channels at any time point; largest mean absolute amplitude across time at any channel; and largest mean absolute amplitude across time and channels. Histograms were plotted of the four values, and thresholds for trial rejection specified manually based on the point where the upper tail deviates from a normal distribution. Epochs were rejected if any of their four values exceeded its threshold, and ∼10% of trials were rejected for each subject. Visual inspection of a subset of epoch waveforms confirmed that this method removed bad epochs successfully. Surviving epochs were averaged within their respective stimulus conditions, followed by low-pass filtering at 35 Hz.

Based on evoked peak topographies observed in pilot experiments, FCz was chosen as the sole channel from which to present time-domain data, and three time windows were determined that maximally captured the three deflections characterizing the evoked response. The mean evoked potential within each time window was taken as the basis for statistical analysis.

Because we had no hypothesis about MMN latency, the duration of MMN responses was relatively long, and there were no clear differences in MMN latency, we did not subject MMN latency to any formal analysis. The primary outcome measure was MMN amplitude, with MMN-timeframe response magnitudes to standards, and N1 and P50 magnitudes as secondary outcome measures.

##### Statistical analysis.

Statistical analysis was performed in MATLAB. On account of Lillefort's test not indicating more datasets deviating from a normal distribution than expected by chance, ANOVA was used as the basis for statistical analysis. For comparison of chronic tinnitus subjects and controls, a three-way ANOVA with full interaction terms was applied, with group (tinnitus or control), frequency (edge or center), and intensity (low or high) as the factors of interest. As the acute tinnitus group was not matched to a control group, it was subject to a two-way ANOVA, with interaction term, with frequency and intensity as the factors of interest. The simulated tinnitus group was subject to a two-way ANOVA, with interaction term, with state (“tinnitus” on or off) and intensity as the factors of interest. Each ANOVA was performed separately on standards and deviants (deviants minus standards). The receiver-operator characteristic (ROC) curve was generated using standard MATLAB functions.

## Results

### Subject characteristics

Subject groups comprised 26 unselected chronic tinnitus subjects, 26 age- and hearing-matched controls, 15 acute tinnitus subjects, and 20 healthy controls studied with and without the simultaneous presentation of simulated tinnitus based on tinnitus subjects' psychophysical tinnitus matches. Their characteristics, along with their individual tinnitus matches and derived stimulus parameters, are summarized in Table 1. Due partly to prioritizing audiometric matches at the stimulus frequencies, there were significant differences between chronic tinnitus and matched control groups in sex and stimulus intensity. The latter may have reflected the matching procedure or (appropriate) compensation for hyperacusis in the tinnitus group. Stimulus loudness at the tinnitus edge frequency was, on average, higher in the control than chronic tinnitus group (Table 1), but overlap between groups was high. To ensure that this did not lead to spurious results, we repeated the primary analysis after excluding the 6 tinnitus subjects with the lowest stimulus intensities (in dB SL) at the edge frequency; this balanced the group means for the edge frequency stimulus intensity (41.0 vs 42.7 dB SL, *p* = 0.75) and increased the statistical significance of the main finding (discussed in its respective section) from *p* = 0.0009 to *p* = 0.0001. The inclusion of unselected tinnitus subjects, including those with severe high-frequency hearing loss, older volunteers, and both tonal and narrowband noise types of tinnitus, potentially may have added noise and variance to the data, but we considered this inclusivity important to prove the applicability of any findings to the broader tinnitus population as opposed to a particular subset.

### Spatiotemporal organization of stimulus response

**Figure 3.**
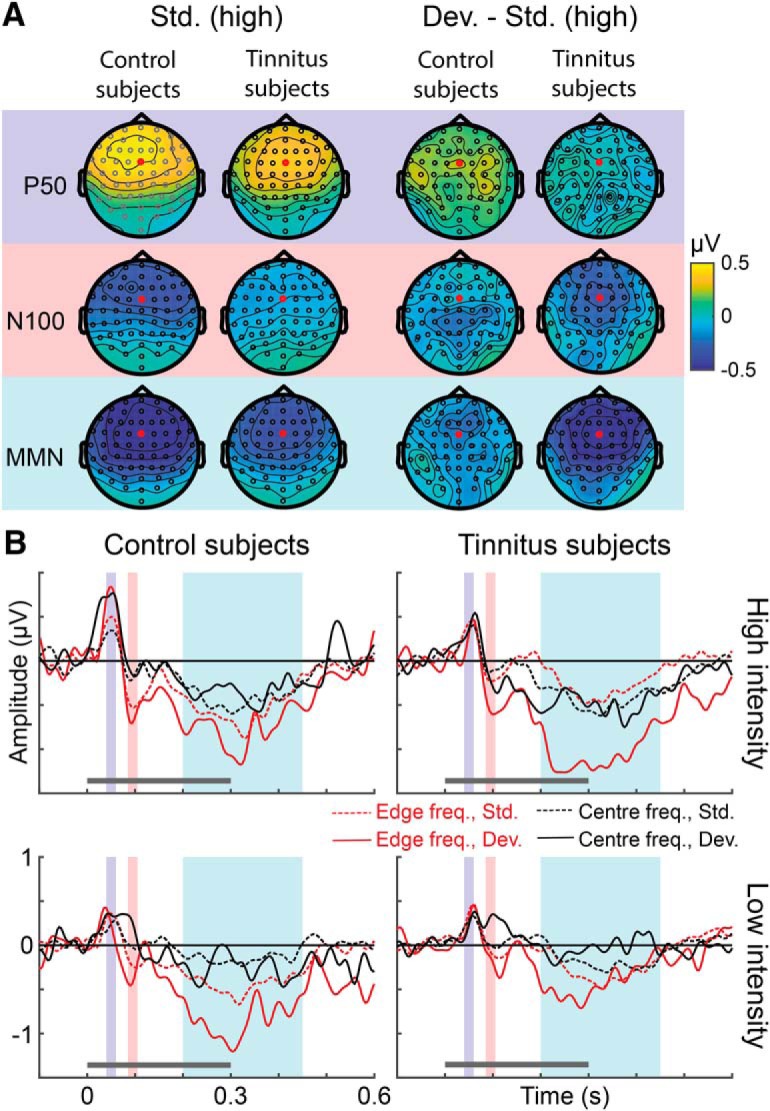
Spatiotemporal distribution of evoked responses. ***A***, Group mean scalp topographies within color-coded time windows capturing the three dominant waveforms. Illustrative responses are shown for the high-intensity standards, and upward deviant minus high standard responses, only. Std., Standard.; Dev., deviant. ***B***, Evoked waveforms from the FCz electrode, highlighted in red in ***A***, to both standard (dashed) and intensity deviant (solid) stimuli at the tinnitus center (black) and edge (red) frequencies. Gray horizontal bars represent stimulus presentation. Colored vertical bars represent the color-coded time windows corresponding to P50, N100, and MMN, as shown in ***A***, and forming the basis of statistical analyses.

In a roving MMN paradigm (Fig. 1), with isochronous 300 ms pure tones matched to either the center frequency or lower edge (subjectively defined as “just outside” the tinnitus sound) of the tinnitus frequency band as the stimuli, and stimulus intensity as the roved parameter, the event-related potential was characterized (Fig. 3) by approximately equally sized P50 and N100 responses, and a prolonged late negative potential, peaking at 200–450 ms in keeping with the timeframe of MMN. This is long for MMN in general, but we note that the one study to examine intensity deviants in tinnitus (Mahmoudian et al., 2013) showed later responses to these than other deviants, in keeping with what we observed here. Furthermore, it is recognized that smaller perceptual changes are associated with later MMN responses (Näätänen and Alho, 1995).

### Early auditory evoked potentials (P50 and N100) are unaffected by tinnitus

There were no differences in standard or deviant P50 responses related to subject group, stimulus frequency, or stimulus intensity. In a three-way ANOVA (subject group, stimulus frequency, and stimulus intensity), N100 response magnitudes to standard stimuli showed a main effect of larger responses to high-intensity stimuli (*p* < 0.05), which was an expected and trivial finding. An equivalent analysis of deviant-minus-standard responses showed a main effect of larger responses to the tinnitus edge (lower) than tinnitus center (higher) frequency (*p* < 0.005). The lack of differences between tinnitus and control groups in these early responses makes simple acoustic differences in stimuli, such as loudness, an unlikely explanation for the tinnitus-related changes described below.

### Tinnitus-irrelevant duration deviants

To exclude broad differences in predictive processes not specifically related to tinnitus or the underlying hypothesis, we incorporated occasional shorter 150 ms stimuli to serve as duration deviants. Duration deviants, in the absence of intensity changes, elicited clear MMN responses, which showed no significant differences on account of stimulus frequency or intensity, or subject group.

### Late (MMN timeframe) responses to standard stimuli show small differences due to tinnitus

**Figure 4.**
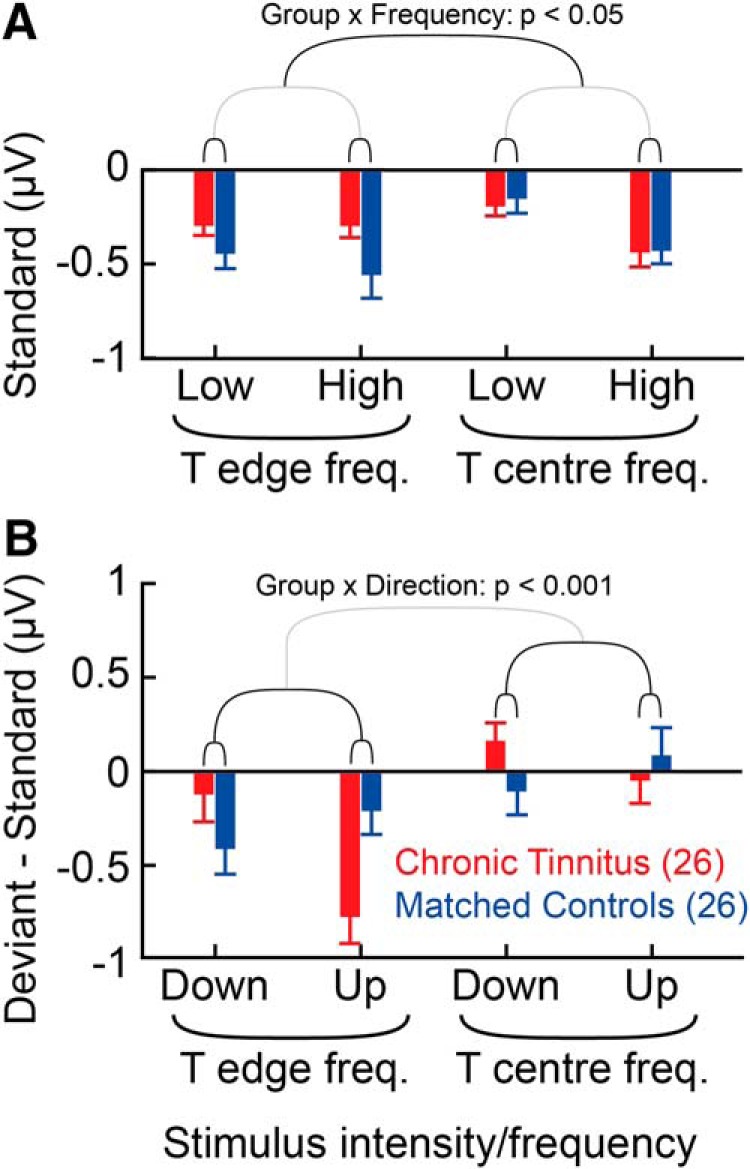
MMN amplitudes in chronic tinnitus and control subjects. Error bars indicate group mean ± SE. Color coding represents subject group: Red represents chronic tinnitus. Blue represents matched controls. ***A***, MMN timeframe responses to standard stimuli. ***B***, MMN responses to deviant minus standard responses. Significant differences relevant to tinnitus status are indicated by *p* values and nested brackets. Black brackets represent variables, or interactions, associated with significant effects based on ANOVA. Gray brackets represent variables not significantly contributing to effects. The core finding was a significant (*p* < 0.001) group (chronic tinnitus vs control) × direction (upward vs downward) interaction in deviant MMN responses.

Standard responses in the MMN time window showed a main effect of being larger for high- as opposed to low-intensity stimuli (*p* < 0.005), which was expected. As shown in Figure 4*A*, there was a group × frequency interaction (*p* < 0.05), whereby the control group, but not the tinnitus group, had larger responses to the lower (edge) frequency standards.

### Asymmetry of MMN responses to intensity deviants differentiates tinnitus subjects from controls

There was a main effect of larger deviant minus standard responses to the lower (edge) than higher (center) frequency (*p* < 0.0001). This mirrored the equivalent difference seen in the N1 responses to deviants, and is likely to be for the same reason. As shown in Figure 4*B*, our principal finding was in line with our hypothesis, in that there was a group × direction interaction (*p* < 0.001), whereby tinnitus subjects had larger responses to upward deviants (minus standards), whereas matched controls had larger responses to downward deviants (minus standards). The effect, which we term Intensity Mismatch Asymmetry (IMA), applied to both tinnitus center and tinnitus edge frequencies; but in keeping with the main effect of frequency, the absolute effect appeared larger at the edge frequency, although the contribution of frequency to this interaction was not statistically significant. Repeating the analysis after excluding the 6 tinnitus subjects with the least intense edge frequency stimuli (to balance mean intensity with the control group) showed the same finding, but with the greater level of statistical significance of *p* = 0.0001.

**Figure 5.**
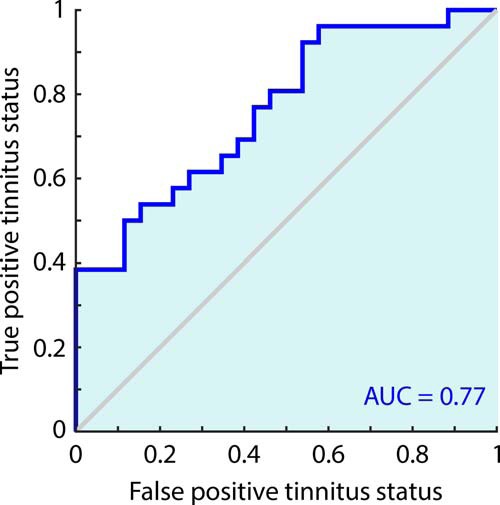
ROC curve for classification of subjects as chronic tinnitus or matched control.

To assess whether IMA can serve as a biomarker for tinnitus, we used the simple metric (averaged across stimulus frequencies) of upward deviants (minus standards) minus downward deviants (minus standards). Figure 5 shows the ROC curve for this metric, which results in an area under the curve of 0.77, indicating the favorable end of “fair” diagnostic accuracy. We aimed to avoid the use of a more complicated classifier metric, which might have produced greater accuracy, to constrain our findings to those that might be ported directly to animal studies. No significant linear correlations were observed between this metric and either THI or visual analog scale loudness score.

### Tinnitus-related MMN changes are present in the acute, as well as chronic, stages

We have previously hypothesized that there is a window of reversibility following initial tinnitus onset, before the tinnitus prediction becomes pervasive, although the length of this window would be unclear, potentially ranging from a scale of days to months. In a two-way ANOVA (intensity and frequency), the group of 15 subjects with new-onset tinnitus (usually within the past 3–4 weeks) showed a main effect of upward intensity deviants yielding larger MMN responses than downward deviants (*p* < 0.05).

### Absence of equivalent changes in simulated tinnitus

The above finding of IMA could theoretically be for the reason hypothesized, that the aberrant prediction responsible for tinnitus skews sensory predictions, or for the more trivial, but still diagnostically useful, reason that the presence of any quiet continuous sound alongside the stimuli skews predictions toward that quiet intensity. To distinguish these possibilities, we conducted the same experiment in 20 healthy controls, with and without hearing loss, with half the blocks containing the addition of simulated tinnitus based on tinnitus subjects' matching data. To maintain a sufficient number of trials, only stimuli at the tinnitus center frequency were used. No significant differences were found between the “tinnitus on” and “tinnitus off” state; in a two-way ANOVA featuring deviant direction (up or down) and state (on or off), the *p* values for main effects of state and state × direction interaction were 0.81 and 0.77, respectively.

## Discussion

### IMA differentiates tinnitus subjects from controls

We tested the hypothesis that development of chronic tinnitus requires formation of a pervasive “default” prediction of a (usually quiet) constant sound within a specific frequency band, and that this prediction favors perceptual recognition of tonotopically specific spontaneous firing in the auditory pathway as a real sound (i.e., tinnitus) rather than ignoring as noise (Sedley et al., 2016). Processing of auditory stimuli within or close to the relevant frequency band might be altered by skewing of all predictions toward the characteristics of the default prediction (Fig. 1). We hypothesized that these skewed predictions would be detectable in MMN responses to intensity deviants around the tinnitus frequency; because downward deviations in intensity involve stimuli becoming quieter, therefore, more similar to the default prediction, tinnitus subjects would show reduced response magnitudes. Conversely, upward deviations in intensity involve stimuli becoming louder; hence, more distant from the default prediction, tinnitus subjects would show increased response magnitudes. Thus, we hypothesized that contrast between upward and downward intensity deviants might serve as an objective marker of tinnitus, and our results support this hypothesis in both the acute (at ∼4 weeks from onset) and chronic stages of tinnitus.

### The IMA effect reflects tinnitus specifically

In theory, differences in MMN responses might occur simply because of an ongoing sound filling in the interstimulus gaps, in which case IMA would be an epiphenomenon of tinnitus, rather than a causative factor. To differentiate these possibilities, we studied 20 nontinnitus controls with and without the simultaneous presentation of narrowband noise derived from subjects' tinnitus matches. Short-term simulated tinnitus should not alter default predictions of the kind hypothesized to underlie tinnitus because: (1) we only presented it for ∼60 s at a time, which would not be a long enough timescale to form pervasive default predictions; and (2) not everybody would necessarily change their default prediction, even after a sufficiently long duration.

The addition of simulated tinnitus within this second control group did not produce any appreciable change in MMN responses, suggesting a specific role in tinnitus.

### Mechanisms potentially underlying IMA

Auditory MMN is generated by a bilateral network of primary and nonprimary auditory cortex and inferior frontal gyrus (Garrido et al., 2008). Reciprocal interaction between these centers is argued to comprise the bottom-up propagation of prediction errors, which signal discordance between prior prediction and sensory input, and the top-down updating of sensory predictions in light of this new evidence (Garrido et al., 2008, 2009), although other explanations include sensory memory, local adaptation to a stimulus, and change detection (Garrido et al., 2009). MMN amplitude is also sensitive to higher-level statistical structure in stimulus sequences (Garrido et al., 2013), and therefore also provides a quantitative indication of the improbability of a stimulus based on prior predictions. Ventrolateral PFC (including inferior frontal gyrus) has been argued to form part of a “tinnitus core” network, which also includes auditory, inferior parietal, and parahippocampal cortex (De Ridder et al., 2014b). Parahippocampal cortex has shown altered resting-state activity contralateral to the tinnitus ear (Vanneste et al., 2011) and resting-state fMRI correlation with auditory cortex (Maudoux et al., 2012; Schmidt et al., 2013), and based on its prominent role in auditory memory is a potential source of persistent auditory predictions. While these networks are likely contributors to the IMA effect we observed here, as the present study does not provide source-resolved activity, it does not in itself specify the brain basis of the effect. Future work might address this issue with imaging modalities with higher spatial resolution.

MMN magnitudes might be affected by changes in central gain, including related to hyperacusis, or deficient noise cancellation via frontostriatal gating, which amounts to a gain control mechanism (Leaver et al., 2011; Rauschecker et al., 2015). P50 suppression is often used as a marker cortical input gating (Yadon et al., 2009) and might have been expected to be a sensitive marker of any gating changes if present. However, there were no differences in any evoked response magnitudes to standard stimuli between tinnitus and control groups, suggesting against a straightforward gain or hyperacusis-related explanation. There are, however, more nuanced aspects of gain, such as dynamic range adaptation, and so we cannot altogether rule out changes in gain in the broader sense as a contributory factor.

We attempted to standardize attention by having all subjects watch a subtitled movie they found engaging, and all subjects claimed they were able to largely ignore the auditory stimuli and attend to the movie. However, this was not formally quantified; hence, some differences between groups cannot be ruled out. Similarly, subjects with substantial tinnitus-related distress might attend more to auditory stimuli or perceive intensity increases in a more threatening way. However, we observed no correlation between magnitude of IMA effect and THI score.

### Previous MMN studies in tinnitus

Previous MMN and equivalent studies (Weisz et al., 2004; Holdefer et al., 2013; Mahmoudian et al., 2013; Asadpour et al., 2018; El-Minawi et al., 2018; Mohebbi et al., 2019b) have varied according to the type of deviant, the paradigm used, control matching for hearing loss, and, importantly, whether stimulus frequencies were standardized or targeted to subjects' tinnitus. Studies with nontargeted stimulus frequencies have reported slightly smaller MMN responses to deviants of all types tested (Holdefer et al., 2013; Mahmoudian et al., 2013), and minor differences in P300 oddball responses to auditory and visual stimuli (Asadpour et al., 2018). At the audiometric (not tinnitus) edge frequency, tinnitus patients showed larger MMN responses (in the N1 timeframe) to downward frequency deviants than hearing unmatched controls (Weisz et al., 2004), and unchanged responses one octave lower. Frequency deviants, with the deviant at the tinnitus match frequency, and the control frequency 10% different, have been found to be increased compared with controls, with partial resolution of the difference following successful tinnitus retraining therapy (El-Minawi et al., 2018). Using standardized stimulus frequencies at ∼8 kHz (regardless of tinnitus frequency), smaller MMN responses were observed in tinnitus patients with high levels of distress only (Mohebbi et al., 2019b). These studies set a precedent for there being small differences in sensory, mnemonic, and/or predictive processing relevant to the MMN in tinnitus. Our present study is the first to feature intensity deviants targeted to the tinnitus frequency; and as such, our results show a much stronger effect and may provide a way forward for this specific field in tinnitus research.

### Potential use as a biomarker

Successful animal research into tinnitus mechanisms and treatments requires knowing which animals experience tinnitus. Numerous methods have been developed to determine this and broadly fall into two categories. Conditioned behavior models (Rüttiger et al., 2003; Brozoski and Bauer, 2016; Pace et al., 2016) are often regarded as the more accurate, and require lengthy prior training of animals to perform or refrain from certain behaviors, such as licking, during the presence of an ongoing sound. Automatic response methods (Turner et al., 2006; Lobarinas et al., 2013) have the advantage of requiring no training, and exploit involuntary responses, such as the acoustic startle response, in conjunction with stimuli related to the possible tinnitus (e.g., a short gap in an ongoing pure tone) to modify this depending on tinnitus status, but are subject to caveats and controversies (Campolo et al., 2013; Lobarinas et al., 2013), and show inconsistent replicability in humans (Fournier and Hébert, 2013; Shadwick and Sun, 2014; Boyen et al., 2015). The two types of approach have shown limited correlation with each other, and with the presence or absence of an auditory insult potentially sufficient to induce tinnitus (Turner et al., 2006). However, because there is no gold standard in animals against which to test the sensitivity and specificity of a diagnostic tinnitus test, the performance of these measures remains unquantified. Potential biomarkers derived from human tinnitus studies have mainly focused on whole-brain resting-state imaging of electrical activity (Vanneste et al., 2018) or large-scale correlations in cerebral blood flow (Minami et al., 2018; Zimmerman et al., 2019), but these measures are inherently nontransferrable to animals. The IMA technique, as reported here, has the potential to constitute a diagnostic test that is quantifiable in its diagnostic performance, on account of being developed in humans, applicable across species, free from training requirements, and quick to perform. The presence of the IMA effect, even just outside the tinnitus frequency, suggests that its success does not depend upon highly specific tinnitus matching. Another recent study has had the same aim (Han et al., 2017), based on quantifying the acoustic change complex, an evoked response to a change during a stimulus. It yielded only slightly lower ROC performance, but we note that the study was subject to numerous limitations, including only yielding this result at uncomfortably loud stimulus levels, and excluding subjects who were older or had significant hearing loss. Although there are a number of factors to address in follow-up studies (e.g., tuning curves over stimulus frequency and intensity, optimizing stimulus timing and duration, standardized diagnostic cutoffs), we believe the IMA technique might have the potential to serve as a convenient and robust biomarker for future animal studies of tinnitus.

### Parallels with other perceptual disorders

Predictive coding accounts of perception (Rao and Ballard, 1999; Friston and Kiebel, 2009) are popular in neuroscience, and our predictive coding tinnitus model (Sedley et al., 2016) joins other predictive coding models of tinnitus (De Ridder et al., 2014a; Durai et al., 2018), and other pathological perceptual states, including chronic pain (De Ridder et al., 2014a; Hechler et al., 2016; Geuter et al., 2017; Nir and Yarnitsky, 2015), musical hallucinosis (Kumar et al., 2014), psychosis (Adams et al., 2013), and functional neurological disorder (Edwards et al., 2012). These theoretical models generally lack support by measurement of the pathological predictions themselves. Here, we demonstrate proof of concept that pathological predictions can be measured using cheap, widely available tools. As it shares many parallels with tinnitus (Møller, 1997; De Ridder et al., 2011, 2014a; Rauschecker et al., 2015; Vanneste et al., 2018), chronic pain would be a logical condition to extend this approach to next.
